# **Telephone Access Management in Primary Care: Cross-Case Analysis of High-Performing Primary Care Access Sites**

**DOI:** 10.1007/s11606-021-07365-5

**Published:** 2022-02-01

**Authors:** Emmeline Chuang, Amy Bonilla, Susan Stockdale, Aditi Das, Elizabeth M. Yano, Danielle Rose

**Affiliations:** 1 School of Social Welfare, University of California Berkeley, Berkeley, CA USA; 2 Department of Health Policy and Management, Fielding School of Public Health, University of California Los Angeles, Los Angeles, CA USA; 3 HSR&D Center for the Study of Healthcare Innovation, Implementation, and Policy, VA Greater Los Angeles Health Care System, Los Angeles, CA USA

**Keywords:** telephone access, primary care, patient-rated access, qualitative research

## Abstract

**Background:**

Primary care telephone access has been associated with patient satisfaction and emergency department utilization even after accounting for objective appointment wait times. However, relatively little is known about how to best structure and manage telephone access in primary care.

**Objective:**

Assess how primary care telephone access is structured and managed and explore how variation in telephone management may affect primary care teams and patients.

**Design:**

We used 2016 administrative and patient survey data to select six Veterans Administration medical centers (VAMCs) with above-average primary care access (time to third next available appointment) but variable patient-reported access, geographic region, and urbanicity. Semi-structured interviews were conducted August –October 2017.

**Participants:**

Forty-three key stakeholders knowledgeable about primary care, telephone management, and operational priorities nationally and/or within each VAMC.

**Key Results:**

Telephone access was organized and managed differently across sites. Regional call centers were perceived as more efficient but less flexible in tailoring processes to meet local needs. Patient preferences for speaking with their own care teams were cited as a reason to manage telephone access locally rather than regionally, particularly in rural sites. Sites with high patient-rated access described call center functions as well-integrated with primary care team workflow, while those with low patient-rated access perceived telephone management practices as negatively affecting primary care team workload. Call center understaffing was a major barrier to optimal telephone access in all six sites, though rural sites reported greater challenges with provider recruitment and retention.

**Conclusions:**

In VA, efforts to improve telephone access have focused on centralizing call center operations but current call center performance metrics do not account for the extent to which call center functions are integrated with primary care workflows or may impact patient experience. Efforts to improve primary care access should carefully consider impact of telephone management practices on providers and patients.

**Supplementary Information:**

The online version contains supplementary material available at 10.1007/s11606-021-07365-5.

## INTRODUCTION

Improving primary care access is a key focus of many delivery system reform efforts, including the patient-centered medical home (PCMH). Because much of patients’ initial engagement with primary care occurs by telephone, access improvement strategies such as telephone triage often focus on how to better use this technology to facilitate or streamline communication between patients and primary care teams.^1,2^ Telephone access has been associated with patient satisfaction even after accounting for objective appointment wait times,^3^ and barriers to telephone access can contribute to patients’ decision to delay or forgo needed care, or to seek primary care in emergency department settings instead.^4,5^

Despite its importance, relatively little is known about how to best manage telephone access in primary care. The extant literature in this area is often based on practices developed in corporate settings or for use with privately insured populations, raising questions about whether they will be equally effective with hard-to-reach populations or populations with more complex care needs.

The current study contributes to the literature by exploring the role of telephone management practices in efforts to improve access in primary care. Data for this study are drawn from the Veterans Health Administration (VHA), the largest integrated healthcare system in the USA. The VHA emphasizes “open access,” which prioritizes continuity in the patient-care team relationship and in accommodating patient preferences for appointments^6^ and also serves a patient population that on average has poorer health status, higher prevalence of health and behavioral health conditions, and higher medical resource use than the general population.^7,8^

### Primary Care Access in the Veterans Health Administration

In an effort to improve primary care telephone access, in 2016 VHA mandated that all VA medical centers (VAMCs) maintain dedicated telephone contact capabilities in four key areas: primary care appointment scheduling, daytime nurse triage, telephone operating services (to help route patient calls), and pharmacy (refilling or renewing prescriptions). VAMCs had flexibility in deciding how to organize telephone access (local level, regional level, or hybrid) but were required to meet specific performance standards for primary care telephone contact (e.g., 30 s or less for average speed of answer, <5% call abandonment rate).

Preliminary evidence suggests that VAMCs responded to this mandate by making tremendous changes to how telephone access was organized and managed, particularly related to appointment scheduling.^9^ However, a 2017 expert panel on access management within VA continued to identify telephone access as a barrier to Veterans’ ability to access needed care even when appointment slots were technically available.^10^ In particular, expert panel members highlighted a need for continued improvement in Veterans’ experience with telephone systems for making appointments and relaying messages to their primary care providers/care teams on a timely basis. Less clear were specific, actionable tools and strategies for improving telephone functioning.

To our knowledge, prior research has not assessed variation in how primary care telephone access is organized and operated, or explored how differences in telephone management practices may affect primary care teams and/or patients. The current study addresses this gap by drawing on data from key stakeholder interviews with healthcare system leaders to explore variation in primary care telephone access organization and management and perceived impacts on primary care teams and patients.

## METHODS

A multiple case study design was used, with medical centers as the unit of analysis. We restricted our eligible study sample to VAMCs that performed above average in terms of time to third next available appointment (≤ 6 days compared to the national average of 10 days) despite high growth in patient enrollment (i.e., ≥5% growth in number of enrollees in FY 2015 and 2016). Time to third next available appointment (TNAA) reflects how long a patient may need to wait before a primary care appointment becomes available and is widely used by healthcare systems as an indicator of primary care access.^11–13^ Data on TNAA were drawn from the 2017 Patient Aligned Care Teams Compass Facility Performance Summary, which regularly reports facility-level data on primary care access, continuity, and quality as part of the VA’s audit-and-feedback performance monitoring system.^14–16^ Of 152 VAMCs, 66 were identified as above average in TNAA.

We then purposely selected six VAMCs that varied in patient-rated access. Patient-rated access was based on the percentage of patients that responded “Always” to the following question from the Consumer Assessment of Health Plans (CAHPS)– Patient Centered Medical Home items included in 2016 VA Survey of Healthcare Experiences of Patients (SHEP): “In the last 6 months, when you contacted this provider’s office to get an appointment for care you needed right away, how often did you get an appointment as soon as you needed?” SHEP CAHPS is administered monthly to a stratified random sample of patients with an outpatient visit at a VA clinic; sampling weights adjust for proportionate population representation and survey non-response by age, gender, and site size.^17^ In 2016, the overall response rate was 41%. Sites were considered as having high patient-reported access if they were ≥90^th^ percentile on this measure (*n*=18) and low patient-rated access if ≤25^th^ percentile (*n*=8). We then purposively selected three sites with high patient-rated access and three sites with low patient-rated access (hereafter referred to as sites A-F) that also varied in urbanicity (four rural, two urban) and geographic region (each site located in a different VA service network).

Regional and local medical center directors were emailed to introduce the study, inquire about willingness to participate, and identify key stakeholders knowledgeable about primary care access, local telephone management practices, and local operational priorities. Table 1 provides an overview of the sites in our sample. Within each of these 6 sites, we interviewed between 5 and 11 key stakeholders, including local healthcare directors or executive committee members, primary care chiefs and/or nursing leaders, administrative services leaders, human resources managers, telephone contact or “call center” managers, and, when appropriate, regional directors and call center leads. To better understand national VHA policies related to primary care telephone management, we also interviewed three key stakeholders from VA OVAC. Key stakeholders received an email invitation to participate in the study, followed by a maximum of three additional contacts (telephone or email). All except three key stakeholders consented to be interviewed (*n*=43 of 46; response rate 93%).

**Table 1.** Characteristics of Participating Medical Centers (*n*=6)

	**Site A**	**Site B**	**Site C**	**Site D**	**Site E**	**Site F**
**Organizational context**						
Patient-reported access^1^	High	High	High	Low	Low	Low
Urbanicity	Rural	Rural	Urban	Rural	Rural	Urban
Number of primary care patients^2^	20–25K	<10K	20–25K	20–25K	20–25K	>75K
Prior experience with call center?	10 years	<1 year	10 years	2.5 years	2.5 years	8 years

^1^Patient-reported access based on 2016 SHEP survey data, with sites identified as high-performing if ≥90^th^ percentile on the measure and as low-performing if ≤25^th^ percentile

^2^Number of unique primary care patients was drawn from 2017 Patient Aligned Care Teams Compass Facility Performance Summary data.

Interviews were conducted by telephone August–October 2017 by a trained interviewer. Interview questions addressed how primary care telephone access was organized, including use of call centers (i.e., a centralized location where telephone calls were answered or made) for primary care appointment scheduling and nurse telephone triage; how telephone access was managed (e.g., staffing, performance metrics, processes for communicating with other departments); perceived effects of telephone management practices on primary care; and facilitators and barriers to primary care telephone access (see Appendix). To provide context for potential effects of telephone management practices on primary care, participants were also asked about how open access scheduling functioned at their facility and current strategic priorities in primary care.

On average, interviews lasted ~45 min. With participants’ permission, all except one interview was recorded. For this one interview, notes were used in place of a recording at the participant’s request. All recordings were transcribed verbatim by a professional transcriptionist and subsequently reviewed for accuracy by a member of our research team. Final transcripts and interview notes were imported into the qualitative software NVivo 11.0 (QSR International) for analysis. We used template analysis, in which an initial codebook informed by key domains of interest in the interview guide was refined to incorporate emergent themes in the data, e.g., patient preferences.^18^ All transcripts were coded by two investigators. Discrepancies in coding were discussed until consensus was reached. Coded data were used to generate case studies summarizing general themes within each code for each site. Case studies were then reviewed to identify general themes as well as differences between sites with high vs. low patient-rated access. We also explored potential differences between sites located in urban vs. rural areas.

This study was deemed exempt from Institutional Review Board review at the authors’ institutions because it was conducted with approval of VA Central Office for quality improvement purposes.

## RESULTS

### Organization of Telephone Access at the Local or Regional Level

Sites varied considerably in how telephone services were organized (Table 2). All six sites reported at least some reliance on call centers for primary care scheduling and nurse telephone triage, and on an automated phone service for pharmacy refills and renewals. However, in three sites, telephone services were managed at the local medical center level; in one site, all primary care scheduling and nurse telephone triage was managed at the regional level; and in the remaining two sites, a “hybrid” approach was used, in which scheduling was managed locally while other telephone services such as nurse triage were “in-sourced” to the regional level.

**Table 2.** Select Telephone Access Organization and Management Practices at Participating Medical Centers (*n*=6)

	**Site A**	**Site B**	**Site C**	**Site D**	**Site E**	**Site F**
Centralization at local or regional level?	Hybrid^1^	Hybrid^1^	Local	Local	Regional	Local
**Primary care appointment scheduling**						
Who is responsible?	Local call center	Shared local call center^2^*and* PC teams	Local call center	Local call center	Regional call center	Shared local call center *and* PC teams
Do patients have direct telephone access to primary care teams?	Yes	No	No^3^	Yes	No	Yes
**Nurse telephone triage**						
Who is responsible?	Regional call center	Regional call center *or* PC nurses	Local call center *or* PC nurses	Local call center	Regional call center *or* PC nurses	Local call center *or* PC nurses
Able to schedule?	No	No	Yes	Yes	No	No
**Call center staffing**						
Number and type of call center staff on-site	13 MSAs	0^2^	11 MSAs; 4 nurses	6 MSAs; 6 nurses	14 MSAs^4^	17 MSAs; 10 nurses

^1^Hybrid call centers include a mix of locally and regionally managed functions

^2^Centralized call center operated and located at another local VAMC

^3^Patients had direct telephone access to primary care teams until approximately 4 months before the interviews were conducted

^4^Regional call center staff responsible for primary care scheduling are hired by the medical center and located on-site but managed by a regional call center manager

*PC*, primary care; *MSA*, medical support assistant

Differences in how telephone access was organized were not associated with patient-rated access or with sites’ location in an urban or rural area. Instead, key informants in all sites reported substantial disagreement regarding whether call center functions should be centralized (i.e., regionally managed) or decentralized (i.e., locally managed). In general, centralization of call center functions was described as more efficient and allowing for greater standardization of practices and ability to manage unexpected surges in local call volume. By contrast, local management of call centers was perceived as preferred by patients and as allowing for greater flexibility in tailoring call center procedures to support local primary care needs, e.g., to accommodate specialized scheduling processes for part-time providers or teams providing care to patients experiencing homelessness.

*“* *I came from a VA where we managed the call center internally and… here, because [our regional service network] manages the call centers… there are greater restrictions on what the call center can and cannot do that can cause grief for both staff and patients... There needs to be more flexibility of ‘one size does not fit all’ to respond to Veteran needs.” -Director*

### Patient Access to Primary Care Teams

In half of sites, patients did not have direct access to primary care teams (i.e., only call center staff were permitted to transfer the call). In addition, none of the regional nurse telephone triage centers was able to directly schedule patients for needed appointments, limiting their ability to achieve first-call resolution. At these sites, patients were described as preferring to circumvent the nurse triage line in favor of leaving a message for subsequent call-back from their primary care nurse. Neither practice was associated with patient-rated access as measured by the VA SHEP survey but both were identified by respondents as a source of patient frustration with call centers.

*“* *We don’t send a lot of calls to the [regional] nurse triage line because our patients… don’t want to talk to a nurse sitting in [another city] when they don’t have any idea who that person is or that person doesn’t know them. A lot of them are hesitant to talk to the triage line.” – Call center manager*

### Call Center Staffing

All six sites reported relying on frontline workers such as medical support assistants (MSAs) to support all call center functions except telephone triage, which was typically handled by registered nurses. Staffing practices did not appear to be linked with patient-rated access or with site location in an urban or rural area. Instead, all six sites also identified chronic understaffing of call centers as a major barrier to effective telephone management. Understaffing of call centers was attributed to a wide range of factors, including rapid increase in new patients, high MSA turnover rates, lengthy hiring and onboarding processes, and regional staffing shortages. In two sites, lack of leadership support for primary care was identified as resulting in understaffing of not only call centers, but also primary care in general.

High turnover within the call centers, which was identified as a concern in five of the six sites, was primarily attributed to call center staff being rapidly promoted to better positions elsewhere within the organization or departing for higher paying jobs within the community. When asked about strategies for sustaining call center functioning despite high turnover, one site described hiring part-time staff to provide coverage during peak call volume hours and ensure a steady supply of qualified staff. When asked about retention strategies, site C (the only site not experiencing high MSA turnover) described implementing flexible work practices such as compressed workweek and telework after 6 months of sustained employment.

*“When you have telework and add a compressed work schedule, it helps with work-life balance... They work an extra hour every day but then get an extra day off every pay period… That’s a great incentive for staff.”* *-HR director*

### Call Center Performance Metrics

All call centers reported focusing on VHA-mandated performance metrics related to call volume, average speed to answer (ideally 30 s or less), and call abandonment rates (ideally <5%). In some sites, call center supervisors also reported informally monitoring average “talk time” with one site reporting a goal of having staff handle calls “within two and a half minutes per call.” By contrast, primary care staff were more interested in call center impacts on veteran satisfaction (“the end user of all this”) and on quality of communication with the call center, which was perceived as directly impacting their workload. However, only two of six sites reported collecting data specific to veteran experience with telephone access and no sites described holding call center staff accountable for quality or nature of follow-up communication with primary care teams.

Cross-case comparisons revealed that self-reported performance on VHA-mandated call center metrics was not related to patient-rated access, but communication between call center staff and primary care teams mattered. In sites with low patient-rated access sites, call center staff were encouraged to communicate with primary care teams primarily via notes or alerts in the electronic health record (EHR), while in sites with high patient-rated access also reported robust use of less formal communication mechanisms (e.g., secure instant messaging or telephone).

### Patient Dissatisfaction with Call Centers as a Barrier

Key informants at all sites also identified patient dissatisfaction with call centers as a concern, due to strong preference among many patients for speaking with their “own” nurses and “own” primary care teams. In the three sites that had more recently transitioned to a call center model (<3 years), patient dissatisfaction was perceived as highest immediately after the transition, but as persisting to some degree over time, particularly among patients located in rural areas and those for whom trust was a concern. At several sites, key informants noted that patient preferences for having their needs met same-day by their primary care teams would choose to “walk-in” to clinics to get their concerns addressed rather than speak with a call center staff member or wait 72 h to be called back by a primary care team member. Walk-ins are considered particularly disruptive of workflows and burdensome on primary care teams. Several respondents expressed frustration with patient preferences for speaking with local staff for appointment scheduling or telephone triage rather than using centralized call centers or online scheduling.

*“* *If I could wave a magic wand and have our patients realize they don’t need to be talking to the clinic where they have their care to get their appointment scheduled, that would be wonderful… [Right now] the veterans think they need to be talking to somebody at their clinic.”* *-Director*

### Perceived Effects of Telephone Management Practices on Primary Care

Key informants at all six sites agreed that use of call centers to streamline appointment scheduling, prescription refills, and triage helped reduce telephone wait time and overall volume of calls fielded by primary care teams. As one respondent put it, “The sheer volume of calls… It’s unclear how many MSAs per clinic you’d need to pick up the phone if [call centers] didn’t exist.” However, sites varied in whether other anticipated benefits of call centers were realized. For example, at site A, call centers were viewed as “expediting right calls getting to the right place” and as saving time in ways that allowed primary care team members work to the top of their license. By contrast, at site F, the call center was described as reducing overall call volume and workload for nurses and MSAs, but resulting in increased work for providers due to the high volume of EHR alerts and notes generated by call center staff. Similarly, at site C, informants described a contentious relationship between call center staff and primary care, with call center staff perceiving themselves as not only “a gatekeeper between patients and primary care teams” but a “dumping ground [for the hospital]” and primary care teams expressing dissatisfaction with call center staff “taking shortcuts” and “kicking more complex issues to primary care” in an effort to ensure call center performance metrics were met.

Cross-case comparison revealed that sites with high patient-rated access scores had more fully integrated interdisciplinary team members such as call center staff and pharmacists into primary care team work processes. For example, in site D (low patient-rated access), key informants reported limited integration of pharmacists in the care team. By contrast, site A (high patient-rated access) had licensed independent pharmacists stationed with primary care teams, which meant that call center staff could forward relevant calls directly to pharmacists “without needing to tie up the [primary care] provider.”

Similarly, while key informants in all sites identified opportunities for improvement in the extent to which call center processes were integrated with primary care teams’ workflow, low-performing sites were more likely to emphasize ways in which call center processes negatively impacted primary care. For example, key informants at site F (low patient-rated access) identified multiple ways in which current processes for managing telephone access negatively affected primary care team workload, such as triage nurses putting notes in the EHR about patients needing follow-up appointments in primary care rather than directly transferring the patient to centralized scheduling to get an appointment scheduled. Similarly, key informants at site E (low patient-rated access) described “inflexible” standardized regional call center procedures as obstructing local efforts to improve primary care, e.g., by failing to accommodate a request to have both providers and nurses (rather than just providers) flagged in the EHR when patients requested opioid prescription refills so that nurses could first talk to the patient.

### Differences Between Urban and Rural Sites

Four of the sites in our sample were located in rural communities. Compared to their urban counterparts, key informants in rural sites cited greater difficulty recruiting providers and staff, particularly specialist providers. Key informants at rural sites also reported greater investment in telehealth and other innovative technologies designed to reduce patient travel time and improve their facilities’ ability to serve as a one-stop shop for care. Finally, key informants in these sites all emphasized patients’ desire to maintain “local” connections as a major consideration in determining whether and how to consolidate call center functions.

*“* *In a small facility, it’s helpful when staff knows their patients… Pushing things out to call centers, especially when you have a high turnover rate, you lose the sense of community. Patients like the sense of community.” –Key stakeholder, Rural site*

## DISCUSSION

This qualitative study contributes to the literature by exploring the extent to which telephone access management practices varied across six medical centers that were considered high-performing in availability of primary care appointments but varied in patient-rated access. We explored the perceived impact of cross-site differences in telephone access organization and management on primary care teams, and also sought to identify factors that differentiated medical centers with high vs. low patient-rated access scores.

We found that while most medical centers relied on centralized call centers for primary care scheduling and nurse triage, call centers varied substantially in how they were organized and in the scope of call center responsibilities. While these differences did not appear to be directly linked to patient-rated access scores, in general regionally managed call centers were perceived as more efficient, while locally managed call centers were perceived as more responsive to patient preferences and variation in local needs. The difficulty of balancing system-level efficiency considerations with patient-centric practices was voiced by all sites in our sample, and consistent with another recent study on access improvement initiatives in the VHA.^9^ In our study, patient preferences for more personalized telephone access were perceived as being particularly salient in rural communities and in working with populations for whom trust in the healthcare system may be of concern. When patient preferences for telephone access were not met, patients were described as choosing to walk in for same-day appointments to address non-urgent concerns instead, defeating the initial intent of the call center as a mechanism for improving access while reducing burden on primary care teams.

Study findings also suggest the importance of carefully considering not only call center efficiency and patient preferences but overall system context when deciding how telephone access should be managed. In our study, medical centers with high patient-rated access had more fully implemented patient-centered medical home principles and reported higher quality communication between call center staff and primary care teams. Key informants at these centers were also less likely to describe current call center processes as negatively impacting primary care. Current call center performance metrics assess department-specific metrics such as average wait time and first call resolution, but not coordination with other departments, the extent to which call center processes are integrated with primary care workload in ways that improve overall system functioning, or whether patients are satisfied with telephone services received.

Several limitations should be considered in interpreting findings from the current study. First, most key informants in our sample were based in VA medical centers, all of whom serve predominantly male patient populations; thus, study findings may not reflect experiences of stakeholders providing or receiving care in other settings. Second, our study sample included only medical centers with above-average primary care access in terms of appointment availability (an important indicator of primary care capacity) and may therefore underestimate the potential impact of telephone access management practices on primary care teams and patients. Finally, our study does not include direct patient perspectives, though we asked key informants to speak to patient perceptions and when available, patient satisfaction data and other patient feedback collected within participating medical centers.

Despite these limitations, our study highlights the importance of telephone management practices in efforts to improve primary care access and the need for further research that more systematically identifies current practices and their impact on primary care teams and patients. Particularly following the COVID-19 pandemic, an increasing number of primary care access improvement initiatives rely on use of telephone, email, and other technology to facilitate communication between patients and members of the primary care team.^19^ As the use of these technologies increases, there is a need to better understand factors that influence effective integration of such technologies into primary care teams’ workflow, or that influence access to care for patient populations that may be “hard-to-reach” or that have more complex care needs. Extant evidence suggests that not all providers or patients are equally ready to utilize telehealth, with small primary care practices, safety net providers, or socioeconomically and medically disadvantaged patient populations most likely to experience barriers to effective use.^20,21^ Careful attention is needed to ensure that increased use of these technologies does not exacerbate existing inequities in access and quality of care.

## Supplementary Information

ESM (DOCX 20.1 KB)
