# Supplementary material for: Telephone Access Management in Primary Care: Cross-Case Analysis of High-Performing Primary Care Access Sites
Source: J Gen Intern Med. 2022 Feb 1;37(8):1963–9. doi: 10.1007/s11606-021-07365-5 (PMC8806007; doi:10.1007/s11606-021-07365-5)
Supplement: Supplementary file 1 — (DOCX 20.1 KB) [file 11606_2021_7365_MOESM1_ESM.docx]

**Appendix. Call Center Interview Guide**

*Note: Specific questions asked varied by respondent role.*

***Respondent role***

1. **Can you tell me a little bit about yourself**?
   - What is your role within the VA? How long have you been in this role?
   - How long have you been with the VA?

***Organizational context***

1. **Can you tell me a little bit about how open-access scheduling works at your facility?**
   - What happens when patients call and ask to be seen? Who do they talk to?
   - Who determines how urgent the need is and whether to schedule for primary care or redirect to urgent care or emergency care?
   - Do primary care clinics see “walk-in” patients?
   - Are patients required to see their assigned primary care provider (PCP)? Can they see other providers without their PCP being penalized for continuity issues?
2. **In your opinion, how supportive has regional or local facility leadership been about efforts to improve primary care access?** What gives you this impression?
3. **Is your facility participating in any initiatives to improve access to primary care for Veterans?** [If yes], can you tell me a little bit more about these initiatives?

***Call center organization and structure***

1. **Can you tell me a little bit about how call centers are organized / operated**?
   - Are call centers organized regionally or locally?
   - Are call centers located “off-site” or co-located with the clinics being served?
   - Are there separate call centers for scheduling vs. triage?
   - What are the call center hours of operations?
   - Are any self-service capabilities offered by telephone (e.g., automated prescription refills) before patients are connected to staff?
   - Are there situations when calls may be routed to unattended phones (e.g., no answer or voicemail)? Can you please elaborate on when or why this might occur?
2. **What activities are call centers responsible for at your site?**
   - Can call center staff directly schedule appointments? Anytime or only “after hours”?
   - Do call center staff provide advice re: general medical issues?
   - Do call center staff proactively patients to schedule follow-up appointments or make appointment reminders?
   - How are “new” patients (i.e., those not already assigned to a PCP) handled?
3. **I’d like to learn more about the call center’s role in scheduling appointments in primary care**.
   - Do processes for scheduling appointments differ by service?
   - Any accommodations based on patient characteristics, e.g., differences in scheduling processes for special populations such as homeless veterans or women veterans?
4. **What performance metrics are call centers responsible for?**
   - What type of data are being collected?
   - Do you have access to any of these metrics?
   - How useful do you find these metrics in assessing call center performance?
   - Which (if any) metrics are the most important? Why?
   - Do these metrics cause any perverse incentives / unintended consequences?

***Call center staffing***

1. **How is your call center staffed?**
   - Do call center staff typically work full-time or part-time? If part-time in the call center, what are their other responsibilities?
   - Who do call center staff “report” to?
   - Are staff assigned to specific “services” or functions?
   - **From your perspective, are there adequate staff dedicated to call center functions?**
   - What factors influence your ability to recruit and retain call center staff?
2. **What type of training do call center staff receive?**
3. **What types of supports do call** **center staff have access to?**
   - Do staff have access to on-site or “on-call” consulting providers or pharmacists? What about access to patient’s medical record?
4. **How standardized are call center staff responsibilities?**
   - Are there standardized protocols that staff are expected to follow and complete before closing out a call? If yes, how consistently are these protocols followed?

***Call center integration with primary care and other departments and services***

1. **Is there any overlap in call center functions and PACT team functions?**
   - How frequently do veterans directly call PACT teams?
   - How are PACT team needs handled when the call center is not the first point of contact?
2. **To what extent (if any) do call center staff responsibilities overlap with those of providers/staff in primary care or other “services”?**
   - If present, how is this overlap addressed?
3. **How do call center staff typically communicate with other departments or services**?
   - How are calls and communications with other departments typically documented?
   - Is there a standardized process for communication between call center and clinical staff?
4. **To what extent are interdisciplinary leaders for primary care involved in call center operations?** Are there regular meetings? Do call center leaders participate in PACT operations/steering committee meetings?

***Call center impact***

1. **In what ways does the call center affect your department or service, specifically?**
   - What are the priority issues in your department?
   - In what ways (if any) do call center operations affect your department’s ability to achieve these priorities? What about access to care for Veterans overall?

***Lessons learned and opportunities for improvement***

1. **Is your facility currently engaged in any initiatives to improve call center performance?** Can you tell me a little bit more about these initiatives?
2. **If you could redesign the call center any way you wanted, what would it look like**? E.g., in an ideal world
3. **Can you identify any best practices or lessons learned that might be useful to other VAMCs or VISNs considering how to best improve call center operations?**

***Conclusion***

1. Is there anything I haven’t asked that you think is important for me to know?
